# Association of hypertension with the severity and fatality of SARS-CoV-2 infection: A meta-analysis

**DOI:** 10.1017/S095026882000117X

**Published:** 2020-05-28

**Authors:** Jingqi Zhang, Jing Wu, Xiaohua Sun, Hong Xue, Jianguo Shao, Weihua Cai, Yang Jing, Ming Yue, Chen Dong

**Affiliations:** 1 Department of Epidemiology and Statistics, School of Public Health, Jiangsu Key Laboratory and Translational Medicine for Geriatric Disease, Medical College of Soochow University, Soochow, China; 2 Soochow University Hospital, Soochow University, Soochow, China; 3 Nantong Third People's Hospital, Nantong, China; 4 Department of Infectious Diseases, The First Affliated Hospital of Nanjing Medical University, Nanjing, China

**Keywords:** COVID-19, fatality, hypertension, SARS-CoV-2, severity

## Abstract

Hypertension is a common comorbidity in COVID-19 patients. However, the association of hypertension with the severity and fatality of COVID-19 remain unclear. In the present meta-analysis, relevant studies reported the impacts of hypertension on SARS-CoV-2 infection were identified by searching PubMed, Elsevier Science Direct, Web of Science, Wiley Online Library, Embase and CNKI up to 20 March 2020. As the results shown, 12 publications with 2389 COVID-19 patients (674 severe cases) were included for the analysis of disease severity. The severity rate of COVID-19 in hypertensive patients was much higher than in non-hypertensive cases (37.58% *vs* 19.73%, pooled OR: 2.27, 95% CI: 1.80–2.86). Moreover, the pooled ORs of COVID-19 severity for hypertension *vs.* non-hypertension was 2.21 (95% CI: 1.58–3.10) and 2.32 (95% CI: 1.70–3.17) in age <50 years and ⩾50 years patients, respectively. Additionally, six studies with 151 deaths of 2116 COVID-19 cases were included for the analysis of disease fatality. The results showed that hypertensive patients carried a nearly 3.48-fold higher risk of dying from COVID-19 (95% CI: 1.72–7.08). Meanwhile, the pooled ORs of COVID-19 fatality for hypertension *vs.* non-hypertension was 6.43 (95% CI: 3.40–12.17) and 2.66 (95% CI: 1.27–5.57) in age <50 years and ⩾50 years patients, respectively. Neither considerable heterogeneity nor publication bias was observed in the present analysis. Therefore, our present results provided further evidence that hypertension could significantly increase the risks of severity and fatality of SARS-CoV-2 infection.

## Introduction

Coronavirus disease 2019 (COVID-19), a newly emerging acute respiratory disease, is caused by severe acute respiratory syndrome coronavirus 2 (SARS-CoV-2) and mainly transmitted via respiratory droplets [1]. Compared with SARS-CoV and MERS-CoV, SARS-CoV-2 is well capable of sustained community transmission, with each case estimated to infect another 2−3 uninfected persons on average [2]. As of 3 April 2020, approximate one million COVID-19 cases and 40 000 deaths have been reported in more than 200 countries/regions around the world [3]. Therefore, it is urgently needed to identify the risk factors associated with the severity and fatality of SARS-CoV-2 infection.

Recently, several clinical studies have noticed that hypertension was one of the most common comorbidities in patients with SARS-CoV-2 infection [4–6]. For example, in one cohort of 191 patients from Wuhan, China, Zhou *et al*. reported that about half of the patients had different comorbidities, with hypertension being the most common (30%) [7]. In a cohort of 138 hospitalised patients with COVID-19, Wang *et al*. observed that hypertension (31.2%), diabetes (10.1%), cardiovascular disease (14.5%) and malignancy (17.2%) were the most common coexisting medical conditions [8]. In addition, an analysis of an outpatient and inpatient cohort of 1099 patients with COVID-19 showed that 15% of the patients had a history of hypertension [9]. However, published articles with different study designs and populations usually yield different estimates and effect sizes. Therefore, a comprehensive and systematic analysis is necessary because of this variability.

As one of the most important public health problems in the 21st century, hypertension affects more than one billion individuals worldwide [10]. However, the influence of hypertension on SARS-CoV-2 infection remains unclear. Therefore, we conducted the present meta-analysis to evaluate the associations of hypertension with the severity and fatality of SARS-CoV-2 infection. This assessment might be helpful to further understand the characteristics of SARS-CoV-2 infection in hypertensive patients.

## Methods

### Search strategy and selection criteria

This meta-analysis was conducted in accordance with the ‘Preferred Reporting Items for Systematic Reviews and Meta-Analyses (PRISMA)’ statement [11]. Articles published up to 20 March 2020 in PubMed, Elsevier Science Direct, Web of Science, Wiley Online Library and CNKI were considered for this review. We searched the medical subject heading (MeSH) terms of ‘2019-nCoV’, ‘NCIP’, ‘COVID-19’ or ‘SARS-CoV-2’ and ‘hypertension’. Additionally, we performed manual searches on the references of selected articles to avoid omission.

**Fig. 1.**
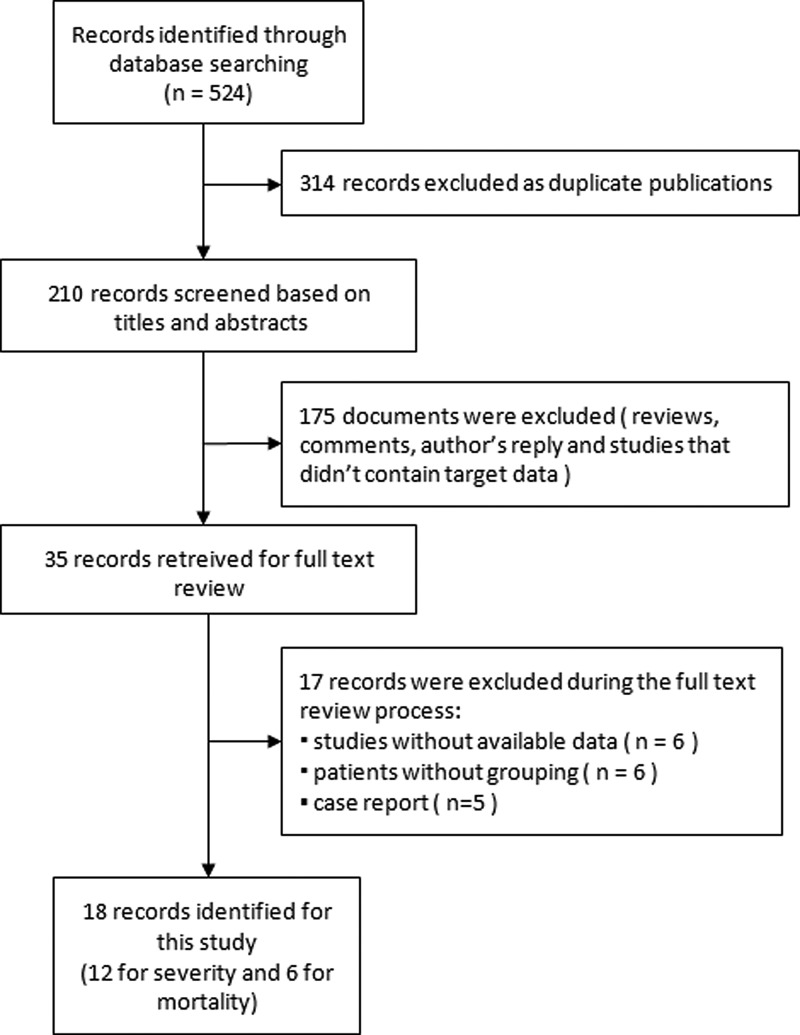
Flow diagram of the studies included in the meta-analysis.

To be included in this meta-analysis, the study had to: (1) COVID-19 cases: diagnosed according to the ‘New Coronavirus Infected Pneumonia Diagnosis and Treatment Program (5th version)’ [12]. All of the included COVID-19 cases should be laboratory confirmed hospitalised patients on the basis of positive qRT-PCR results for SARS-CoV-2 in swab samples; (2) clinical features: definite disease severity or fatality according to the ‘New Coronavirus Infected Pneumonia Diagnosis and Treatment Program (5th version)’ [12]; (3) parameters: clear information about hypertension; (4) study population: at least included 10 cases. Case reports, reviews, meta-analysis articles, letters, comments, editorials, expert opinions, studies without adequate information, and the articles published in languages other than English and Chinese were excluded. The detail retrieval process was shown in Figure 1.

### Definitions

The types of COVID-19 severity was determined according to the ‘New Coronavirus Infected Pneumonia Diagnosis and Treatment Program (5th version)’ [12]. Briefly, the criteria for disease severity determination were as follows: (1) Mild cases: the clinical symptoms were mild, and there was no sign of pneumonia on imaging; (2) Moderate cases: only showing fever and respiratory symptoms with radiological findings of pneumonia; (3) Severe cases (for adults): ①Respiratory distress ⩾30 breaths/min; ② Oxygen saturation ⩽93% at rest; ③ Arterial partial pressure of oxygen (PaO_2_)/fraction of inspired oxygen (FiO_2_) ⩽300 mmHg; (4) Critical cases: ① Respiratory failure and requiring mechanical ventilation; ② Shock; ③ With other organ failure that requires ICU care.

In this study, hypertension was defined as systolic blood pressure greater than 140 mmHg or diastolic blood pressure greater than 90 mmHg, or use of antihypertensive medications, or a self-reported history of hypertension [13].

### Data extraction

Titles, abstracts and full texts were screened independently by two reviewers in the first step (Jingqi Zhang and Jing Wu). Discrepancies in data interpretation were resolved by discussion with a third author (Chen Dong) when necessary. Data extracted from the selected studies included the first author's name, publication date, average age, type of study design and severity of COVID-19 in hypertensive and non-hypertensive patients. All reviewers performed a quality control check between the final data used in the meta-analysis and the original publications to prevent extraction errors.

### Quality assessment

The quality of each included article was assessed using the Newcastle-Ottawa Scale (NOS) based on the following dimensions [14]: COVID-19 cases selection, comparability and outcomes of disease severity or fatality. The cases selection contained four items: (1) the representativeness of the cases; (2) the appropriate determination of severe cases; (3) the appropriate determination of non-severe cases; (4) the appropriate determination of hypertension. Comparability contained one item: which was to consider the comparability between patients with and without hypertension when designing and statistical analysis. The lowest quality of the literature was 0 and the highest quality was 9 stars. Assessment scores of 0–3 points, 4–6 points and 7–9 points were considered as poor, fair and good studies, respectively. Simultaneously, the quality of each included study was further evaluated using Strengthening the Reporting of Observational Studies in Epidemiology (STROBE) (2007 version) checklist in this study.

### Statistical analysis

In this study, the mild/moderate and severe/critical COVID-19 cases were classified into non-severe and severe groups, respectively. Forest plots were used to illustrate the severity and fatality of SARS-CoV-2 infection in hypertensive and non-hypertensive patients, which were described by pooled odds ratio (pooled OR, 95% confidence intervals (CI)). The magnitude of heterogeneity between-study was tested using *I*^2^ statistics. *I*^2^ values of 25%, 50% and 75% represented low, moderate and high degrees of heterogeneity, respectively. If there was no evidence of between-studies heterogeneity (*I*^2^ ⩽ 50%), a fixed-effects model was used to calculate the combined OR. Otherwise, a random-effects model was selected [15]. Sensitivity analysis was performed by excluding studies successively. Furthermore, subgroup analysis was performed according to age (<50 or ⩾50 years). In this study, the possibility of publication bias was evaluated by using the visual inspection of a funnel plot and Egger's test. A *P* value of Egger's test <0.10 was considered as an indication of potential publication bias. All analysis was performed using the Package of ‘meta’ in R software (Version 3.6.3), and significance was defined as *P* < 0.05 with the use of a two-sided test unless otherwise specified.

## Results

### Study selection

A total of 929 articles were obtained by the electronic and manual search. Of these, duplicated articles were excluded at the first stage (*n* = 314). Then, 175 studies were removed after screening titles and abstracts. In addition, 17 studies were excluded after thoroughly reviewing the full texts due to the following reasons: studies without available data (*n* = 6), patients were not classified into severe (severe/critical) and non-severe (mild/moderate) groups (*n* = 6), and case reports or other study design (*n* = 5). Finally, 18 studies recorded with 4505 patients were included in this analysis. Figure 1 summarised the flow diagram of the studies selection.

### Study characteristics and methodological quality

As the results shown in Table S1, all included patients were from China, including Hubei [7, 8, 16–23], Jiangxi [24], Beijing [25], Henan [26], Chongqing [27, 28], Anhui [29] and China CDC [5, 9]. Among of them, 12 studies analysed the severity of COVID-19 in hypertensive patients [8, 9, 16–20, 24–28]. Significant associations between hypertension and COVID-19 severity were reported in five studies [8, 9, 18, 19, 24]. Additionally, among studies analysed the fatality of SARS-CoV-2 infection in hypertensive patients [5, 7, 21–23, 29], three studies reported that hypertensive patients carried higher risk of COVID-19 fatality [5, 7, 22].

All included studies were retrospective studies. As the results shown in Table S1, the methodological qualities of the included studies were similar. Nine studies had a moderate risk of participant comparability and confounding variables [8, 9, 16–19, 22, 23, 25]. Six, seven and five studies had 6, 7 and 8 points of assessment score in this analysis, respectively. In addition, no significant difference was observed between the results obtained from NOS and STROBE analysis (Table S2).

### Associations between hypertension and COVID-19 severity

**Fig. 2.**
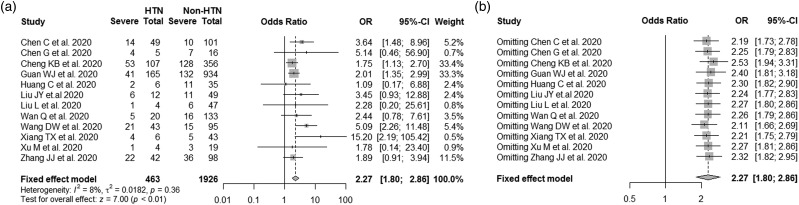
Meta-analysis of the associations between hypertension and COVID-19 severity. (a) Forest plot of the COVID-19 severity for comparison between hypertensive and non-hypertensive patients; (b) Sensitivity analysis of the COVID-19 severity for comparison between hypertensive and non-hypertensive patients after excluding any single study one-at-a-time.

Twelve studies with 2389 COVID-19 patients (674 severe cases) were included for the disease severity analysis. As shown in Table 1 and Figure 2a, the severity rate of SARS-CoV-2 infection in hypertensive patients was much higher than in non-hypertensive cases (37.58% *vs* 19.73%, pooled OR: 2.27, 95% CI: 1.80–2.86). There was no considerable heterogeneity observed between study (*I*^2^ = 8%, *P-heterogeneity* = 0.36). When the individual study was excluded from the analysis one-at-a-time, the pooled ORs ranged from 2.11 (95% CI: 1.66–2.69) when the study by Wang DW *et al*. [8] was excluded to 2.53 (95% CI: 1.94–3.31) when the study by Cheng KB *et al.* [18] was excluded (Fig. 2b). There was neither evidence of publication bias with Egger's test (*P* = 0.13), nor asymmetry indicated by the funnel plot (Fig. S1a).

**Table 1.** Characteristics of COVID-19 severity in the hypertension and non-hypertension groups in identified studies

Author	Age	Gender Male (%)	Hypertension (*n*)	Non-hypertension (*n*)	Hypertension/COVID-19 *n* (%)		Non-hypertension/COVID-19 *n* (%)	
					Non-severe	Severe	Non-severe	Severe
Xiang TX *et al*.	42.9 (18.0–78.0)	33 (67.3%)	6	43	2 (33.33)	4 (66.67)	38 (88.37)	5 (11.63)
Chen G *et al*.	56.3 (42.0–70.6)	17 (81.0%)	5	16	1 (20.00)	4 (80.00)	9 (56.25)	7 (43.75)
Wan Q *et al*.	46.0 (10.0–74.0)	77 (50.3%)	20	133	15 (75.00)	5 (25.00)	117 (87.97)	16 (12.03)
Wang DW *et al*.	56.0 (42.0–68.0)	75 (54.3%)	43	95	22 (51.16)	21 (48.84)	80 (84.21)	15 (15.79)
Zhang JJ *et al*.	57.0 (25.0–87.0)	71 (50.7%)	42	98	20 (47.62)	22 (52.38)	62 (63.27)	36 (36.73)
Liu L *et al*.	45.0 (34.0–51.0)	32 (62.7%)	4	47	3 (75.00)	1 (25.00)	41 (87.23)	6 (12.77)
Guan WJ *et al*.	47.0 (35.0–58.0)	637 (58.1%)	165	934	124 (75.15)	41 (24.85)	802 (85.87)	132 (14.13)
Huang C *et al*.	49.0 (41.0–58.0)	30 (73.0%)	6	35	4 (66.67)	2 (33.33)	24 (68.57)	11 (34.43)
Liu JY et.al	40.0 (1.0–86.0)	31 (50.8%)	12	49	6 (50.00)	6 (50.00)	38 (77.55)	11 (22.45)
Xu M *et al*.	46.0 (40.5–52.0)	15 (65.2%)	4	19	3 (75.00)	1 (25.00)	16 (84.21)	3 (15.79)
Cheng KB *et al*.	51.0 (43.0–60.0)	244 (52.7%)	107	356	54 (50.47)	53 (49.53)	228 (64.04)	128 (35.96)
Chen C *et al*.	59.0 (43.0–75.0)	84 (56.0%)	49	101	35 (71.43)	14 (28.57)	91 (90.10)	10 (9.90)

**Fig. 3.**
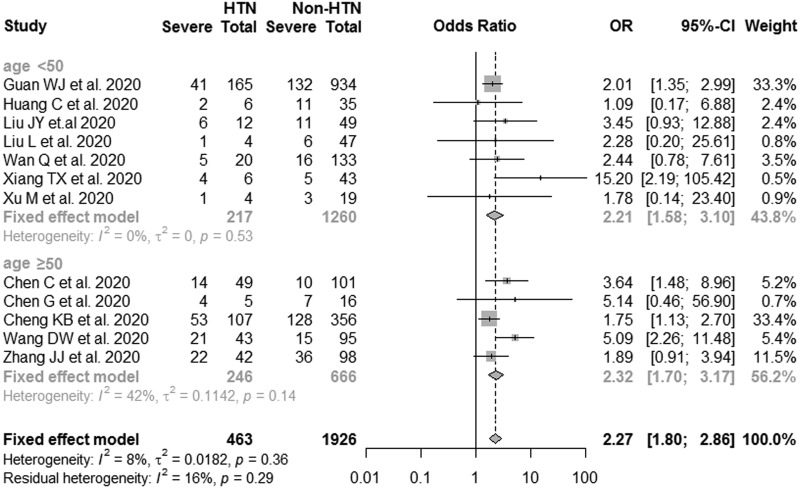
Subgroup analysis of the associations between hypertension and risk of COVID-19 severity (age <50 years and age ⩾50years).

As severity of illness was significantly correlated to age in COVID-19 cases, we performed a subgroup analysis to evaluate the effects of hypertension on the COVID-19 severity in different age groups. As the results shown, the pooled ORs of COVID-19 severity for hypertensive patients *vs.* non-hypertensive cases was 2.21 (95% CI: 1.58–3.10) and 2.32 (95% CI: 1.70–3.17) in age <50 years and ⩾50 years patients, respectively (Fig. 3). No considerable heterogeneity was detected between studies within each group.

### Associations between hypertension and fatality of COVID-19

**Fig. 4.**
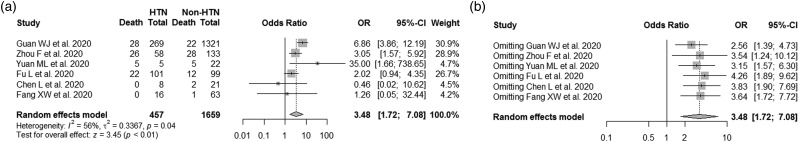
Meta-analysis of the associations between hypertension and COVID-19 fatality. (a) Forest plot of the COVID-19 fatality for comparison between hypertensive and non-hypertensive patients; (b) Sensitivity analysis of the COVID-19 fatality for comparison between hypertensive and non-hypertensive patients after excluding any single study one-at-a-time.

Six studies with 151 deaths among 2116 COVID-19 patients were included in this meta-analysis. As shown in Table 2 and Figure 4a, the fatality rate of SARS-CoV-2 infection in hypertensive patients was 17.72%, which was much higher than in non-hypertensive cases (4.22%). Pooled analysis showed that hypertensive patients carried a nearly 3.48-fold higher risk of dying from COVID-19 (95% CI: 1.72–7.08), with a moderate heterogeneity between-study (*I*^2^ = 56%, *P-heterogeneity* = 0.04). Sensitivity analysis showed that the exclusion of any single study one-at-a-time did not alter the direction or statistical difference (Fig. 4b). Similarly, Egger's test did not suggest the existence of potential publication bias (*P* = 0.67) (Fig. S1b).

**Table 2.** Characteristics of fatality of COVID-19 in the hypertension and non-hypertension groups in identified studies

Author	Age	Male (%)	Hypertension (*n*)	Non-hypertension (*n*)	Hypertension/COVID-19 *n* (%)		Non-hypertension/COVID-19 *n* (%)	
					Non-survival	Survival	Non-survival	Survival
Guan WJ *et al*.	48.9 (32.6–65.2)	904 (57.3%)	269	1321	28 (10.41)	241 (89.59)	22 (1.67)	1209 (91.52)
Zhou F *et al*.	56.0 (46.0–67.0)	119 (62.0%)	58	133	26 (44.83)	32 (55.17)	28 (21.05)	105 (78.95)
Yuan ML *et al*.	60.0 (47.0–69.0)	12 (45.0%)	5	22	5 (100.00)	0 (0)	5 (22.73)	17 (77.27)
Fu L *et al*.	59.6 (50.2–68.1)	99 (49.3%)	101	99	22 (21.78)	79 (78.22)	12 (12.12)	87 (87.88)
Chen L *et al*.	56.0 (26.0–79.0)	21 (72.0%)	8	21	0 (0)	8 (100.00)	2 (9.52)	19 (90.48)
Fang XW *et al*.	45.1 (28.5–61.7)	45 (56.9%)	16	63	0 (0)	16 (100.00)	1 (1.59)	62 (98.41)

**Fig. 5.**
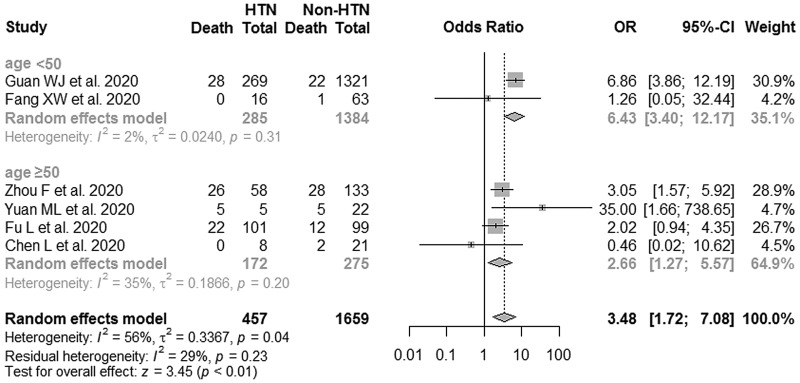
Subgroup analysis of the associations between hypertension and risk of COVID-19 fatality (age <50 years and age ⩾50 years).

Similarly, stratified analysis results further suggested the pooled ORs of COVID-19 mortality for hypertensive patients *vs.* non-hypertensive cases were 6.43 (95% CI: 3.40–12.17) and 2.66 (95% CI: 1.27–5.57) in age <50 years and ⩾50 years patients, respectively (Fig. 5). No significant heterogeneity was presented between studies within each subgroup.

## Discussion

In this study, we reported that the overall rates of severity and fatality were 28.21% and 7.14% in the COVID-19 patients from the included literatures, respectively. Moreover, hypertensive patients with SARS-CoV-2 infection were shown to have 2.27- and 3.48-fold higher risks of severity and fatality compared to the COVID-19 cases without hypertension, respectively. Sensitivity analysis with exclusion of studies one by one did not change the results materially, suggesting that each included study might not have a particularly strong influence on the results. Indeed, the chronic conditions influencing the severity of SARS-CoV-2 such as hypertension, diabetes and obesity have also been addressed in other respiratory illnesses such as MERS, influenza and etc. For example, Alqahtani *et al*. reported that hypertension and diabetes mellitus were prevalent in about 50% of cases of MERS. Moreover, hypertension showed significant association with fatality from MERS-CoV infection (OR: 3.7, 95% CI: 2.02–6.9) [30]. In addition, compared to subjects without hypertension, severe pandemic influenza occurred significantly more often in those who had hypertension (OR: 1.49, 95% CI: 1.10–2.01) [31].

Currently, the exact mechanism of hypertension involvement in severe COVID-19 remains unclear. One potential explanation is the direct injury mediated via angiotensin-converting enzyme 2 (ACE2). Similar to SARS-CoV, a Chinese study recently indicated that SARS-CoV-2 infection was caused by binding of the viral surface spike protein to ACE2 receptor following activation of the spike protein [32]. ACE2 is a monocarboxy peptidase best known for cleaving several peptides within the renin−angiotensin system [33]. Since its discovery in 2000, ACE2 has been considered as a protective factor against increases in blood pressure. Therefore, it is rational to hypothesise that the binding of SARS-COV-2 to ACE2 can reduce the physiological function of ACE2, and then lead to acute adverse outcomes of hypertension such as multi-organ dysfunction [34]. Besides, severale studies have indicated that ACE2 played a critical role in acute lung disease, especially acute respiratory distress syndrome [35–37]. In addition to the heart and lung, ACE2 could be expressed in the intestinal epithelium, vascular endothelium, brain, liver and kidneys [38], providing a potential mechanism for the severity symptoms and multi-organ dysfunction that can be seen with severe SARS-CoV-2 infection.

The epidemiological data of SARS-CoV-2 infection suggested that severe COVID-19 cases were more likely to be older patients with underlying comorbidities (such as diabetes mellitus and hypertension) [5, 7, 9], indicating that age was an important risk factor of severity and fatality of COVID-19. For example, the China CDC recently reported that patients aged ⩾80 years had the highest case fatality rate, 14.8%, among different age groups [39]. Herein, we observed that hypertension was significantly associated with the severity and fatality in SARS-CoV-2 infection in both age <50 years and ⩾50 years groups, suggesting that hypertension could independently increase the risk of disease severity and predict poor outcomes of SARS-CoV-2 infection. Although our present results implied that patients aged <50 years had more fatalities of COVID-19 compared to those patients ⩾50 years, we could not conclude that younger patients are more likely to die of hypertension than older patients because of the paucity of related data. Indeed, further subgroup analysis did not show a significant difference in COVID-19 fatality between patients with and without hypertension in the group consisted of studies with small sample size (references 21, 23 and 29) (pooled OR: 2.82, 95% CI: 0.20–39.15) (Fig S3). However, these results should be carefully interpreted because of the wide confidence interval. Therefore, more studies are needed to characterize the impacts of hypertension on the COVID-19 severity and fatality in future.

Our study has several limitations. Firstly, publication bias is inevitable in any meta-analysis, and some relevant articles might be missed as we only included English- and Chinese-written studies. Secondly, we could not address the potential impact of anti-hypertension medicines although most hypertensive cases might be treated. Thus, it would underestimate the true difference in severity and fatality of SARS-CoV-2 infection between patients with and without hypertension. Thirdly, the estimate found in this study was derived from hospitalised cases. This might introduce a bias in disease severity and fatality. In addition, the overlap of cases could not be completely avoided because the patients in references 5 and 9 were collected from the China CDC, which did not provide the exact hospital information. For this reason, the pooled estimate would never represent the actual pooled outcome data. However, remove references 5 and 9 from our analysis, which had the largest sample size, did not alter the direction or statistical difference of the present results. Fourthly, the sample size included in this meta-analysis was relatively small, thus it would introduce bias in the results. Last, due to most of the included studies were retrospective studies, there may be a bias regarding collecting medical information in a retrospective manner. Therefore, caution should be considered to interpret findings.

In summary, the current results provided further evidence that hypertension could significantly increase the risks of severity and fatality of SARS-CoV-2 infection. Therefore, it is necessary to establish awareness programmes, implement effective preventive policies to improve the outcomes of SARS-CoV-2 infection in persons with hypertension, which should be helpful for the reduction in the global burden of the disease.

## Supplementary material

For supplementary material accompanying this paper visit http://dx.doi.org/10.1017/S095026882000117X. click here to view supplementary material

## References

[1] Craig AT, Heywood AE and Hall J (2020) Risk of COVID-19 importation to the Pacific islands through global air travel. Epidemiology & Infection 148, e71. 32202489 10.1017/S0950268820000710 PMC7113321

[2] Chen TM (2020) A mathematical model for simulating the phase-based transmissibility of a novel coronavirus. Infectious Diseases of Poverty 9, 24. 32111262 10.1186/s40249-020-00640-3 PMC7047374

[3] Coronavirus Outbreak. Available at https://www.worldometers.info/coronavirus/ (Accessed 02 April 2020).

[4] Guo T (2020) Cardiovascular implications of fatal outcomes of patients with Coronavirus disease 2019 (COVID-19). JAMA Cardiology 2020, e201017. 10.1001/jamacardio.2020.1017 PMC7101506 32219356

[5] Guan WJ (2020) Comorbidity and its impact on 1590 patients with Covid-19 in China: A nationwide analysis. European Respiratory Journal 55, 2000547. 32217650 10.1183/13993003.00547-2020 PMC7098485

[6] Chen T (2020) Clinical characteristics of 113 deceased patients with coronavirus disease 2019: retrospective study. British Medical Journal 368, m1091. 32217556 10.1136/bmj.m1091 PMC7190011

[7] Zhou F (2020) Clinical course and risk factors for mortality of adult inpatients with COVID-19 in Wuhan, China: a retrospective cohort study. Lancet 395, 1054–62. 32171076 10.1016/S0140-6736(20)30566-3 PMC7270627

[8] Wang DW (2020) Clinical characteristics of 138 hospitalized patients with 2019 novel coronavirus-infected pneumonia in Wuhan, China. The Journal of the American Medical Association 323, 1061–9. 10.1001/jama.2020.1585 PMC7042881 32031570

[9] Guan WJ (2020) Clinical characteristics of coronavirus disease 2019 in China. The New England Journal of Medicine 382, 1708–20. 32109013 10.1056/NEJMoa2002032 PMC7092819

[10] Rodriguez-Iturbe B, Pons H and Johnson RJ (2017) Role of the immune system in hypertension. Physiological Reviews 97, 1127–64. 28566539 10.1152/physrev.00031.2016 PMC6151499

[11] Moher D (2009) Preferred reporting items for systematic reviews and meta-analyses: the PRISMA statement. PLoS Medicine 6, e1000097. 19621072 10.1371/journal.pmed.1000097 PMC2707599

[12] National Health Commission of the People's Republic of China. Diagnosis and treatment of new coronavirus pneumonitis (trial version 5). Available at http://www.nhc.gov.cn/yzygj/s7653p/202002/3b09b894ac9b4204a79db5b8912d4440.shtml.

[13] Chobanian AV (2003) The seventh report of the joint National Committee on prevention, detection, evaluation, and treatment of high blood pressure: the JNC 7 report. The Journal of the American Medical Association 289, 2560–72. 12748199 10.1001/jama.289.19.2560

[14] Stang A (2010) Critical evaluation of the Newcastle-Ottawa scale for the assessment of the quality of nonrandomized studies in meta-analyses. European Journal of Epidemiology 25, 603–5. 20652370 10.1007/s10654-010-9491-z

[15] Greenland S (1987) Quantitative methods in the review of epidemiologic literature. Epidemiologic Reviews 9, 1–30. 3678409 10.1093/oxfordjournals.epirev.a036298

[16] Chen G (2020) Clinical and immunologic features in severe and moderate forms of Coronavirus Disease 2019. MedRxiv. doi: 10.1101/2020.02.16.20023903.

[17] Zhang J (2020) Clinical characteristics of 140 patients infected with SARS-CoV-2 in Wuhan, China. Allergy. doi: 10.1111/all.14238. 32077115

[18] Cheng KB (2020) Clinical characteristics of 463 patients with common and severe type coronavirus disease 2019. Shanghai Medical Journal. Available at http://kns.cnki.net/kcms/detail/31.1366.r.20200312.1254.004.html.

[19] Chen C (2020) Analysis of myocardial injury in patients with COVID-19 and association between concomitant cardiovascular diseases and severity of COVID-19. Chinese Journal Cardiology 48, E008. 10.3760/cma.j.cn112148-20200225-00123 32141280

[20] Huang C (2020) Clinical features of patients infected with 2019 novel coronavirus in Wuhan, China. Lancet 395, 497–506. 31986264 10.1016/S0140-6736(20)30183-5 PMC7159299

[21] Yuan ML (2020) Association of radiologic findings with mortality of patients infected with 2019 novel coronavirus in Wuhan, China. PLoS One 15, e0230548. 32191764 10.1371/journal.pone.0230548 PMC7082074

[22] Fu L (2020) Influence factors of death risk among COVID-19 patients in Wuhan, China: a hospital-based case-cohort study. MedRxiv. doi: 10.1101/2020.03.13.20035329.

[23] Chen L (2020) Analysis of clinical features of 29 patients with 2019 novel coronavirus pneumonia. Zhong hua Jie He He Hu Xi Za Zhi 43, 203–8. 10.3760/cma.j.issn.1001-0939.2020.03.013 32164089

[24] Xiang TX (2020) Analysis of clinical characteristics of 49 patients with novel coronavirus pneumonia in Jiangxi province. Chinese Journal of Respiratory and Critical Care Medicine 19, 154–160.

[25] Liu JY (2020) Neutrophil-to-lymphocyte ratio predicts severe illness patients with 2019 novel coronavirus in the early stage. MedRxiv. doi: 10.1101/2020.02.10.20021584. PMC7237880 32434518

[26] Xu M (2020) Clinical analysis of 23 cases of 2019 novel coronavirus infection in Xinyang City, Henan Province. Chinese Critical Care Medicine 32, E010–E010.

[27] Wan Q (2020) Analysis of clinical features of 153 patients with novel coronavirus pneumonia in Chongqing. Chinese Journal Clinical Infectious Disease 13, 16–20.

[28] Liu L (2020) Clinical characteristics of 51 patients discharged from hospital with COVID-19 in Chongqing, China. MedRxiv. doi: 10.1101/2020.02.20.20025536.

[29] Fang XW (2020) Clinical characteristics and treatment strategies of 79 patients with COVID-19. Chinese Pharmacological Bulletin 36, 1–7.

[30] Alqahtani FY (2018) Prevalence of comorbidities in cases of Middle East respiratory syndrome coronavirus: a retrospective study. Epidemiology & Infection 147, 1–5. 10.1017/S0950268818002923 PMC6518603 30394248

[31] Mertz D (2013) Populations at risk for severe or complicated influenza illness: systematic review and meta-analysis. British Medical Journal 347, f5061. 23974637 10.1136/bmj.f5061 PMC3805492

[32] Lan J (2020) Structure of the SARS-CoV-2 spike receptor-binding domain bound to the ACE2 receptor. Nature 581, 215–20. 32225176 10.1038/s41586-020-2180-5

[33] Chen LJ (2015) The ACE2/Apelin signaling, microRNAs, and hypertension. International Journal of Hypertension 2015, 896861. 25815211 10.1155/2015/896861 PMC4359877

[34] Tipnis SR (2000) A human homolog of angiotensin-converting enzyme. Cloning and functional expression as a captopril-insensitive carboxypeptidase. Journal of Biological Chemistry 275, 33238–43. 10924499 10.1074/jbc.M002615200

[35] Jia H (2016) Pulmonary angiotensin-converting enzyme 2 (ACE2) and inflammatory lung disease. Shock 46, 239–48. 27082314 10.1097/SHK.0000000000000633

[36] Kaparianos A and Argyropoulou E (2011) Local renin-angiotensin II systems, angiotensin-converting enzyme and its homologue ACE2: their potential role in the pathogenesis of chronic obstructive pulmonary diseases, pulmonary hypertension and acute respiratory distress syndrome. Current Medical Chemistry 18, 3506–15. 10.2174/092986711796642562 21756232

[37] Kuba K (2010) Trilogy of ACE2: a peptidase in the renin-angiotensin system, a SARS receptor, and a partner for amino acid transporters. Pharmacology & Therapeutics 128, 119–28. 20599443 10.1016/j.pharmthera.2010.06.003 PMC7112678

[38] Hamming I (2007) The emerging role of ACE2 in physiology and disease. Journal of Pathology 212, 1–11. 17464936 10.1002/path.2162 PMC7167724

[39] Novel Coronavirus Pneumonia Emergency Response Epidemiology Team (2020) Vital surveillances: the epidemiological characteristics of an outbreak of 2019 novel coronavirus diseases (COVID-19) – China, 2020. China CDC Weekly 2, 113–22. PMC8392929 34594836

