# Supplementary material for: Association of hypertension with the severity and fatality of SARS-CoV-2 infection: A meta-analysis
Source: Epidemiol Infect. 2020 May 28;148:e106. doi: 10.1017/S095026882000117X (PMC7270484; doi:10.1017/S095026882000117X)
Supplement: Supplementary file 1 [file S095026882000117Xsup.zip › S095026882000117Xsup001.docx]

Table S1 Characteristics and Quality assessment of included studies

| Author | Date of Publication | Region | Research Characteristic | Selection | Comparability | Outcome/  Exposure | Quality score(0-9) |
| --- | --- | --- | --- | --- | --- | --- | --- |
| Guan WJ et al.^[5]^ | 02/25/2020 | 31 province/China | retrospective | 2 | 2 | 3 | 7 |
| Zhou F et al.^[7]^ | 03/09/2020 | Hubei | retrospective | 2 | 2 | 3 | 7 |
| Wang DW et al.^[8]^ | 02/07/2020 | Hubei | retrospective | 2 | 1 | 3 | 6 |
| Guan WJ et al.^[9]^ | 02/28/2020 | 30 province/China | retrospective | 3 | 1 | 2 | 6 |
| Chen G et al.^[16]^ | 02/16/2020 | Hubei | retrospective | 2 | 1 | 3 | 6 |
| Zhang JJ et al.^[17]^ | 02/19/2020 | Hubei | retrospective | 2 | 1 | 3 | 6 |
| Cheng KB et al.^[18]^ | 03/12/2020 | Hubei | retrospective | 2 | 1 | 3 | 6 |
| Chen C et al.^[19]^ | 02/25/2020 | Hubei | retrospective | 2 | 1 | 3 | 6 |
| Huang C et al.^[20]^ | 02/15/2020 | Hubei | retrospective | 3 | 2 | 2 | 7 |
| Xiang TX et al.^[21]^ | 03/02/2020 | JiangXi | retrospective | 2 | 2 | 3 | 7 |
| Liu JY et.al.^[22]^ | 02/10/2020 | Beijing | retrospective | 3 | 1 | 3 | 7 |
| Xu M et al.^[23]^ | 02/14/2020 | Henan | retrospective | 3 | 2 | 3 | 8 |
| Wan Q et al.^[24]^ | 02/24/2020 | Chongqing | retrospective | 3 | 2 | 3 | 8 |
| Liu L et al.^[25]^ | 02/20/2020 | Chongqing | retrospective | 2 | 2 | 3 | 7 |
| Yuan ML et al.^[26]^ | 03/19/2020 | Hubei | retrospective | 3 | 2 | 3 | 8 |
| Fu L et al.^[27]^ | 03/13/2020 | Hubei | retrospective | 2 | 1 | 3 | 6 |
| Chen L et al.^[28]^ | 02/19/2020 | Hubei | retrospective | 2 | 1 | 3 | 6 |
| Fang XW et al.^[29]^ | 02/06/2020 | Anhui | retrospective | 2 | 2 | 3 | 7 |
