# Supplementary figures and images for: Association of hypertension with the severity and fatality of SARS-CoV-2 infection: A meta-analysis
Source: Epidemiol Infect. 2020 May 28;148:e106. doi: 10.1017/S095026882000117X (PMC7270484; doi:10.1017/S095026882000117X)

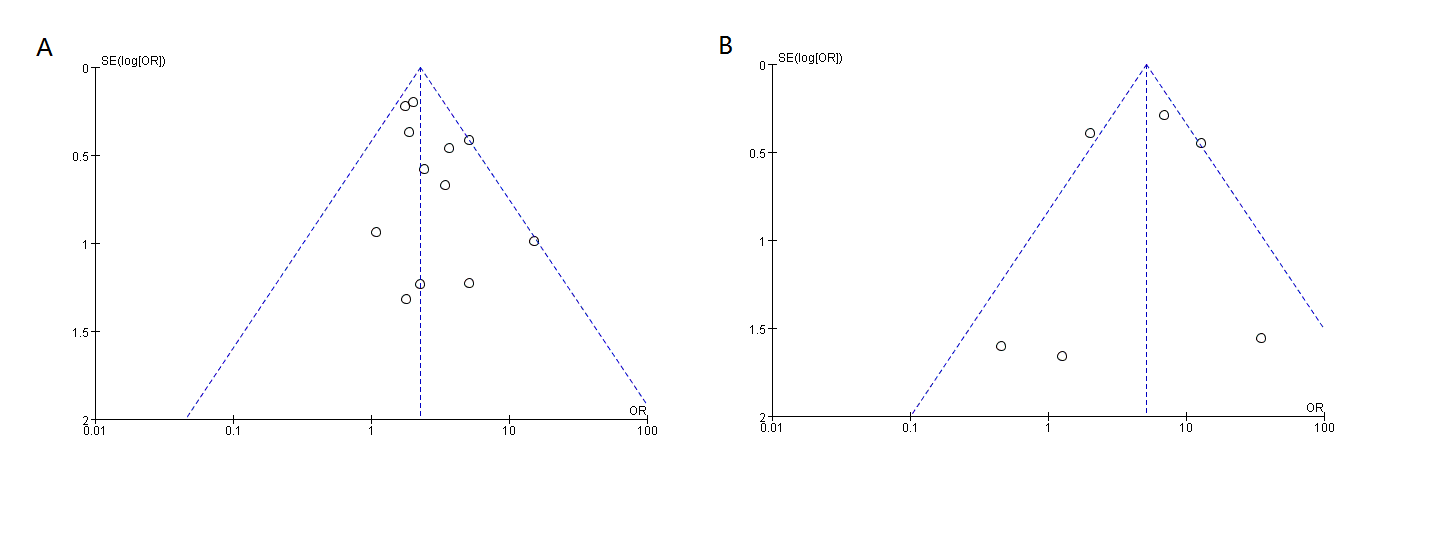

Supplement: Supplementary file 1 [file S095026882000117Xsup.zip › S095026882000117Xsup002.tif]
